# Biodiversity of Forensically Relevant Blowflies (Diptera: Calliphoridae) at the Anthropology Research Facility in Knoxville, Tennessee, USA

**DOI:** 10.3390/insects13020109

**Published:** 2022-01-18

**Authors:** Yangseung Jeong, Lauren M. Weidner, Simon Pergande, Denise Gemmellaro, David E. Jennings, Krystal R. Hans

**Affiliations:** 1 Department of Biology, Middle Tennessee State University, 1301 E. Main Street, Murfreesboro, TN 37132, USA; sp6g@mtmail.mtsu.edu; 2 School of Mathematical and Natural Sciences, New College of Interdisciplinary Arts and Sciences, Arizona State University, 4701 W. Thunderbird Road, Glendale, AZ 85306, USA; lauren.weidner@asu.edu; 3 School of Natural Sciences, Kean University, 1000 Morris Avenue, Union City, NJ 07083, USA; denisucciola@yahoo.com; 4 Vermont Law School, 164 Chelsea Street, South Royalton, VT 05068, USA; david.e.jennings@gmail.com; 5 Department of Entomology, Purdue University, 901 W. State Street, West Lafayette, IN 47907, USA; hans3@purdue.edu

**Keywords:** forensic entomology, biodiversity, Tennessee, Calliphoridae, *Chrysomya megacephala*, *Protophormia terraenovae*

## Abstract

**Simple Summary:**

This study investigates the biodiversity and seasonal distribution of blowflies at the Anthropology Research Facility (ARF) of the University of Tennessee, which is also known as the “Body Farm”. Blowflies are among the first insects that access dead bodies, and have a significant impact on the rate and pattern of decomposition. Although the ARF has been used for numerous taphonomic and entomological studies over the past 40 years, it is not yet fully known what blowfly species are present in the area. After a 14-month-long blowfly survey at the ARF, we collected a total of 3180 adult blowflies, comprising 13 species from 7 genera. *Phormia regina* (Meigen) and *Lucilia coeruleiviridis* (Macquart) were the predominant species collected from this survey, representing 65.9% and 20.6% of collections, respectively. Among the 13 species, *Protophormia terraenovae* (Robineau-Desvoidy) was collected for the first time in Tennessee. In addition to relative abundance, we also investigated blowfly community composition, species abundance, richness, and diversity by season. This research is expected to provide researchers at the ARF with accurate information about the blowflies so that they can plan and design their research accordingly.

**Abstract:**

Understanding the biodiversity and distribution of forensically relevant blowflies (Diptera: Calliphoridae) in a region can aid in legal investigations when insects are associated with remains. For this purpose, we conducted a 14-month-long blowfly survey at the Anthropology Research Facility (ARF) of the University of Tennessee in Knoxville, Tennessee. Traps baited with pork kidney were deployed for 24 h twice a month throughout the study. A total of 3180 adult blowflies were collected, comprising 13 species from 7 genera. *Phormia regina* (Meigen) and *Lucilia coeruleiviridis* (Macquart) were the predominant species collected from this survey, with collections representing 65.9% and 20.6%of total flies captured, respectively. In addition to relative abundance, we investigated blowfly community composition, species abundance, richness, and diversity by season. One state record was identified, with adult *Protophormia terraenovae* (Robineau-Desvoidy) being collected for the first time in Tennessee. Additionally, an earlier record of *Chrysomya megacephala* (Fabricius) in Tennessee was noted. These findings can be used to aid in legal investigations in the area and surrounding areas where work is limited, as well as to provide information on which forensically relevant species should be the subject of future research in the area.

## 1. Introduction

Medicolegal forensic entomology is a discipline that utilizes insects and other arthropods as evidence to help solve legal investigations [1]. Ecologically speaking, a cadaver represents a rich and ephemeral ecosystem that attracts different communities of insects and other arthropods. These insects detect volatile cues released by the decomposing body, which can be used for food, shelter, and as an oviposition substrate [2]. Knowledge of the biology and ethology of these insects, combined with medical and anthropological information, can help answer questions about the circumstances surrounding death. The most important contribution that medicolegal forensic entomology can provide in this regard is the estimation of the minimum time of death (minPMI) by determining the time of colonization (TOC) [3]. In order to use entomological evidence for the estimation of the minPMI, it is necessary to know the entomofauna present in a given geographical area during a specific season, along with information regarding the developmental rate of the major necrophilous species of that area. Diptera—specifically the Calliphoridae family (commonly known as blowflies)—are among the first colonizers of decomposing remains; they have the ability to detect and locate a body within minutes [4] or seconds [5] after its exposure and, therefore, are reliable for the estimation of minPMI.

Worldwide, a number of studies have surveyed calliphorid flies using baited traps or animal carcasses [6,7,8,9,10]. These studies have contributed greatly to our knowledge of blowfly species distribution in different ecoregions. The assemblage of insects to non-human animal remains may vary by geographic region, as demonstrated in Hawaii [11], Virginia [12], British Columbia [4], Australia [13], Switzerland [14], and Thailand [15]. Less research has been conducted using human cadavers, because of practical and legal constraints. Information regarding insects associated with human remains often comes from casework, and is available for British Columbia [16], Hawaii [17], Texas [18,19], Indiana [20], and Italy [21]. Morgues and autopsies conducted at medical institutes can also provide information pertaining to the insects colonizing human remains [22,23,24,25,26,27,28]. Additionally, Matuszewski et al. [29] conducted a global review of the forensic entomology literature pertaining to various animal models when examining whether swine carcasses were appropriate models for humans.

Research institutes with established body donation programs aimed specifically at decomposition-related research are not abundant, but can be found globally (Table 1). These human taphonomy facilities are commonly known as “body farms”, and their main goal is to examine the decomposition of donated human remains in order to analyze thanatological phenomena, including insect activity and colonization [30,31]. The oldest of these facilities is the Anthropology Research Facility (ARF), established in 1980 by Dr. William M. Bass in Knoxville, TN, as part of the University of Tennessee.

Since its inception, the ARF has been used to conduct forensic entomology research using donated human bodies [35,36,37,38,39,40]. The first survey on the insect communities associated with human cadavers was conducted at the ARF in the early 1980s, when four non-embalmed cadavers were exposed in a wooded area to analyze the successional patterns and developmental rates of the necrophilous entomofauna [35]; 10 dipteran families were reported from the survey, but the specimens were not identified at a species level. Although studies rarely capture all species in an area, a species-level analysis on the insect communities of the ARF was performed during the summer of 1998. Shahid et al. [36] reported eight Calliphoridae species associated with three pig carcasses, while Schoenly et al. [41] identified five calliphorid species from a human–pig mixture experiment setting. Even though these efforts could reveal a partial aspect of the entomofauna of the ARF of a specific season, a thorough survey of the forensically relevant blowflies has not yet been conducted. Here, we present the results of a year-round survey of calliphorid flies collected across the ARF using baited traps placed in proximity to decomposing human bodies. This research is expected not only to be used to aid in legal investigations where blowfly-involved human remains are recovered, but also to provide future researchers at the ARF with accurate information about seasonal blowfly activities so that they can plan and design their research accordingly.

## 2. Materials and Methods

### 2.1. Experimental Location and Duration

Flies were collected at the ARF of the University of Tennessee, Knoxville, between March 2018 and April 2019. The area of the location where these traps were placed is ~7000 m^2^. Approximately 100 human body donations were placed at the ARF throughout the period when this research was conducted. The number of bodies placed on the surface at the ARF varied between 2 and 15 per month. The newly placed bodies were in a relatively early stage of decomposition. Including the newly placed bodies, the total number of bodies placed on the surface and exposed to insects at the ARF fluctuated between 74–108 at one time. Geographically, the ARF is located at 35.941046 (latitude), −83.939290 (longitude), which corresponds to the humid subtropical climate zone (Cfa) according to the Köppen–Geiger climate classification [42].

### 2.2. Trap Design and Placement

**Figure 1:**
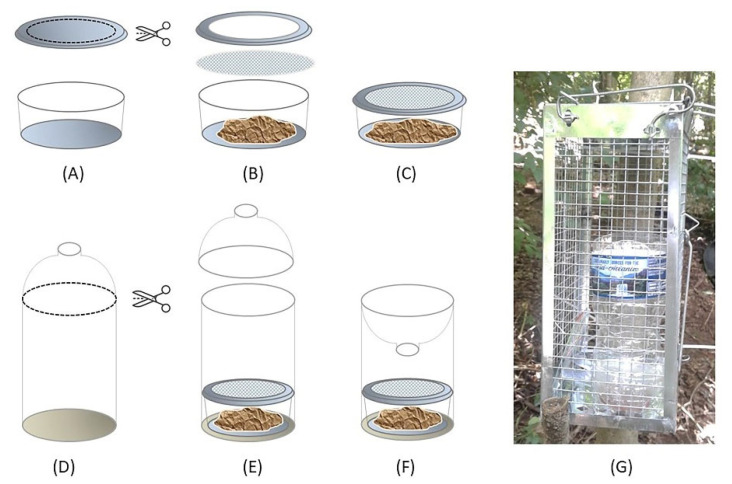
Step-by-step demonstration of the fly trap construction process: (**A**) A large opening is made in the lid of a 59.1 mL portion cup. (**B**) Pork kidney (~20 g) is placed in the cup, and then a piece of 1 mm × 1 mm plastic mesh is placed between the cup and the lid. (**C**) The rim of the open lid clicks with the cup, completing the bait container. (**D**) The top portion of a 500 mL water bottle is cut off. (**E**) The bait container is placed in the bottle. (**F**) The top portion of the bottle is reassembled upside-down. (**G**) The fly trap is placed in the metal rat cage to prevent animal scavenging.

Fly traps were constructed using a transparent 500 mL water bottle, plastic mesh (1 mm × 1 mm), a transparent 59.1 mL plastic portion cup with an open lid, and approximately 20 g of pork kidney, which could fill more than half of the portion cup. The bait (pork kidney) was purchased frozen, but was fully thawed to room temperature by the time of deployment. Traps were designed to use a separate bait container, which had a piece of mesh between the cup and open lid, so that flies could be attracted by the odor of the bait but could not access it for feeding (Figure 1). Traps were originally hung over tree branches ~1.5–2 m above the ground. However, in July 2018, multiple traps were found to have been damaged due to animal scavenging—most likely raccoons [43]. Thus, from August 2018, all traps were placed in metal rat cages (mesh size of 1 cm × 1 cm) that were tied to tree trunks (Figure 1G). The metal cages did not impede flies’ entry into the traps, but effectively prevented scavenging, and no evidence of scavenging was observed through the remainder of the study.

**Figure 2:**
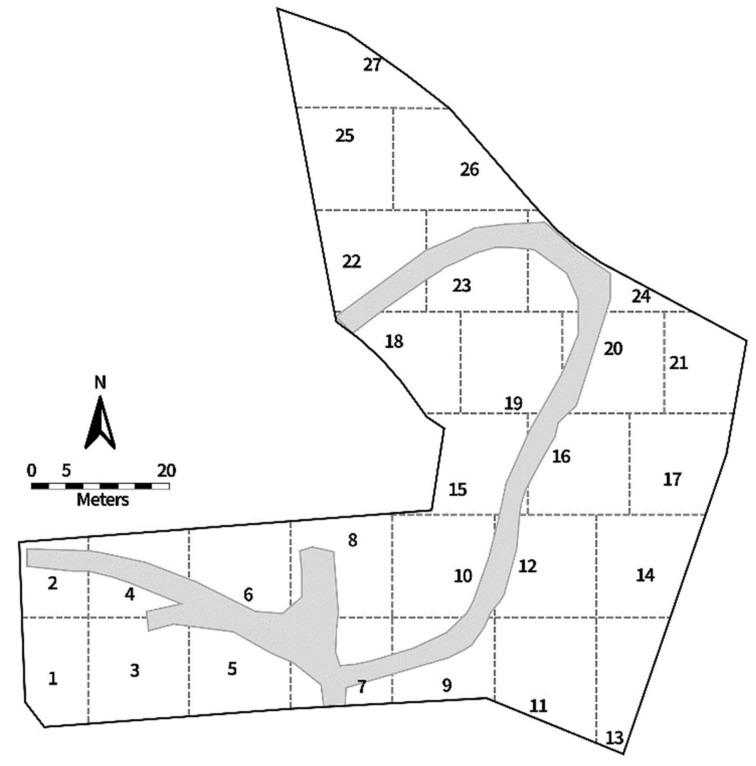
The Anthropology Research Facility (ARF) map, showing 27 sections delineated by dotted lines. The numbers denote the locations of fly traps as well as the section numbers. The gray area is the pathway.

Twenty-seven traps were deployed twice a month during the research period, and all traps remained in the field for 24 h before collection. The traps were deployed in predetermined divided sections throughout the ARF (Figure 2) to capture fly activity throughout the entire area. Traps were located approximately 1–5 m from human remains, depending on body placement and skeletal pick-ups. Traps were approximately 10–20 m apart and, since trap locations were close together, all trap information was pooled for data analysis.

### 2.3. Processing

After collection and visual inspection of traps, all traps containing flies were moved into a laboratory freezer (−20 °C) approximately three hours away from the ARF. To prevent the flies from escaping the traps during trap collection and transportation, the trap openings were temporarily blocked. After being frozen for approximately 24 h, each fly was taken out of a trap, counted, pinned, and assigned a unique identification number. All blowfly identifications were made morphologically using the keys of Jones et al. [44] and Jewiss-Gaines et al. [45]. Specimens are currently stored in the freezer in the Jeong Lab at Middle Tennessee State University.

### 2.4. Temperature Data

A weather station (Model: AcuRite 02064 Wireless Weather Station) was set up in the ARF in March 2018 to collect weather data. The weather station recorded weather conditions every 12 min, including temperature and humidity levels. Additionally, the precipitation data were obtained from Weather Underground (https://www.wunderground.com/weather/us/tn/knoxville, accessed on 23 June 2021), which displays data from a weather station located approximately 1.6 km from the ARF (Table 2).

### 2.5. Data Analyses

We analyzed seasonal variation in blowfly community composition. Seasons were defined as follows: spring (March, April, May), summer (June, July, August), fall (September, October, November), and winter (December, January, February). No flies were collected during the winter months; therefore, that season was not included in the analyses. We calculated the relative abundance, species richness, and Simpson diversity index within each season. Simpson’s index of diversity was calculated as 1-D, where greater values correspond to greater diversity [46].

Before analyzing blowfly community composition, we removed rare species comprising < 1% of the total collection (Table 3), as well as any sample collection periods where the total collection consisted of zero or a single individual. Community composition (based on counts) was initially analyzed using nonmetric multidimensional scaling (NMDS). We then used multiple response permutation procedures (MRPPs) with Bonferroni corrections for multiple pairwise comparisons between seasons [47], followed by indicator species analysis (ISA) [48]. An indicator value (IV) displays the species that is (or are) the best predictor(s) of that season, ranging from 0 (no indication) to 100 (perfect indication). Analyses were conducted using R 4.0.3 [49].

## 3. Results

**Figure 3:**
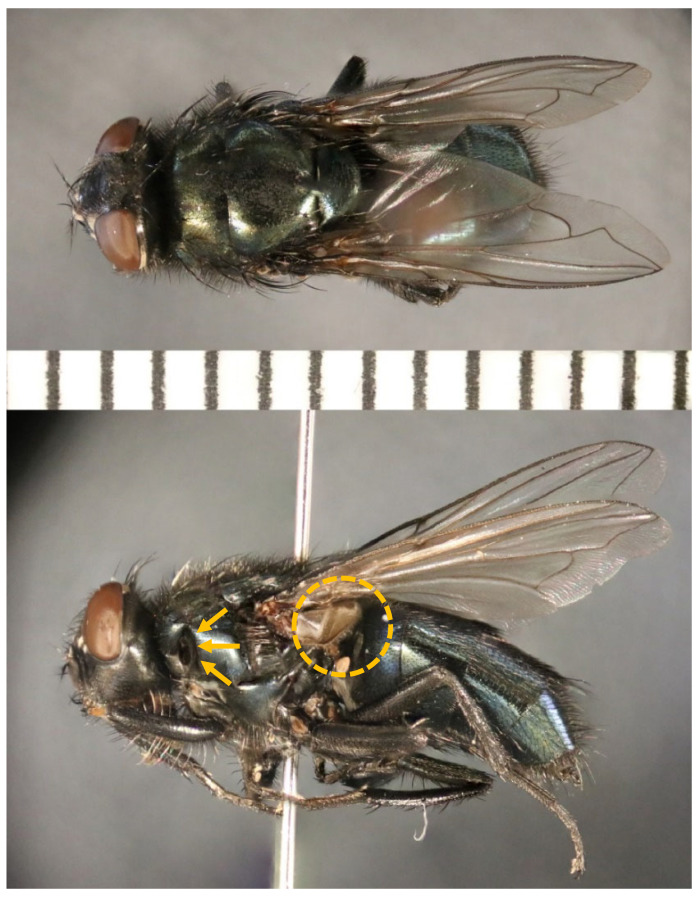
Superior (**top**) and lateral (**bottom**) aspects of *Protophormia terraenovae* (Robineau-Desvoidy) collected from the ARF. Note the black anterior spiracle (arrows) and black hairs on the calypter (circle at the bottom picture). Between the pictures is a 1 mm interval scale.

Of the 3353 flies collected in the traps, 3180 (94.8%) were blowflies, and the remaining 173 (5.2%) were non-calliphorid flies. During the entire study, 13 species of blowflies spanning 7 genera were collected (Table 3). Of this total, 24 individuals were unable to be identified down to the species level due to damage. The dominant species collected throughout the entire study was *Phormia regina* (Meigen), which represented 65.9% of all blowflies, followed by *Lucilia coeruleiviridis* (Macquart) at 20.6%; these were also the dominant blowflies for each season, with *P. regina* being the most common species in spring and summer (78.6% and 64.0%, respectively), and *L. coeruleiviridis* being the most common species in fall (58.9%; Table 3). The species richness was highest in fall (12 species), and did not differ between spring and summer (10 species each), although species composition did (Table 4). Additionally, *Protophormia terraenovae* (Robineau-Desvoidy) was collected for the first time in Tennessee (Figure 3).

**Figure 4:**
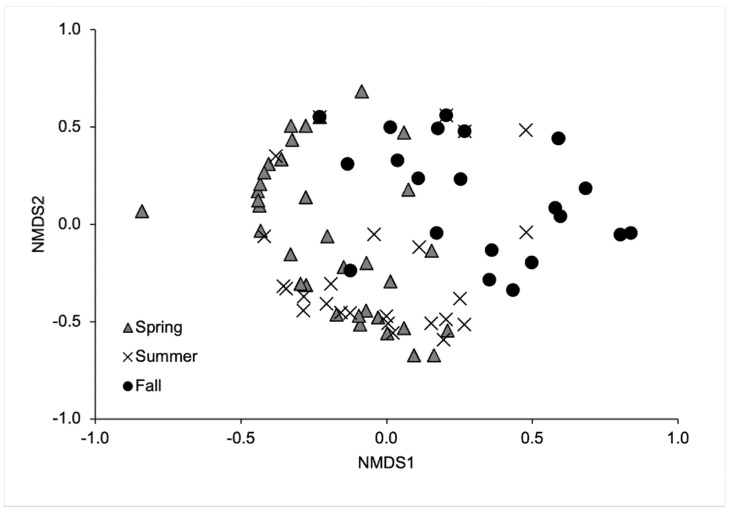
Nonmetric multidimensional scaling ordination of the blowfly population across seasons. This ordination explained 80.9% of the variance (stress = 0.174).

The global model of blowfly communities indicated significant differences between seasons (MRPP: A = 0.078, *p* < 0.001) (Figure 4). Bonferroni-adjusted *p*-values for the pairwise comparisons between seasons showed significant differences in the blowfly communities between spring and fall (MRPP: A = 0.063, *p* = 0.003) and between summer and fall (MRPP: A = 0.092, *p* = 0.003), but not between spring and summer (MRPP: A = 0.025, *p* = 0.051). *Lucilia illustris* (IV = 37.29, *p* = 0.007), *P. regina* (IV = 50.42, *p* = 0.007), and *Pr. terraenovae* (IV = 42.76, *p* < 0.001) were indicators for summer. *Calliphora vomitoria* was an indicator for fall (IV = 19.71, *p* = 0.033). No species were indicators for spring.

## 4. Discussion

This is the first thorough study to examine the seasonal biodiversity of forensically relevant blowflies in the ARF of Knoxville, Tennessee. Thirteen species within seven genera were collected. Extensive surveys of forensically relevant blowflies are not common throughout the United States; however, Weidner et al. [8] conducted a two-year survey of forensically relevant blowflies in New Jersey, with similar findings. For example, *P. regina* was found in all seasons in New Jersey [8], not only agreeing with these findings (when flies were captured) in Tennessee, but also indicating that this species is commonly found throughout the year. Additionally, the two predominant species found in this survey (*L. coeruleiviridis* and *P. regina*) were two of the three main species collected in New Jersey; however, the relative abundance of these species varied between seasons, possibly due to the temperature differences between the two geographical regions. Interestingly, *L. sericata*—the third predominant species in New Jersey—was also collected in Tennessee, but in very low numbers (Table 3); this may be due to the local terrain of the ARF (i.e., naturally wooded area), as 94% of collected *L. sericata* in New Jersey came from urban locations [8]. This survey found that blowfly diversity was highest in the spring (March–May), but the richness was highest during the fall (September–November). Several species were determined to be indicator species for two seasons. This information can be extremely valuable if one is trying to determine a time or season of death for unknown remains that have been severely decomposed. If known indicator species are present, this could provide valuable information about the season of death.

Reed [50] collected 11 species within 5 genera, including *C. livida, C. vicina, C. vomitoria, C. terraenovae, Cy. cadaverina, P. regina, Co. macellaria, L. sericata, L. coeruleiviridis, L. illustris,* and *L. cuprina*. Shahid et al. [37] confirmed the presence of *Ch. rufifacies* at the ARF in the late 1990s. Shahid et al. [36] collected eight species (*C. vicina, Ch. rufifacies, Co. macellaria, L. illustris, Phaenicia (=Lucilia) cluvia, Phaenicia coeruleiviridis, Phaenicia sericata,* and *P. regina*), while Schoenly et al. [41] recorded five calliphorid species (*P. regina, L. illustris, L. sericata, L. coeruleiviridis,* and *Co. macellaria*). Our findings overlapped with 11 of these previously collected species. *Calliphora terraenovae* collected by Reed [50] and *L. cluvia* collected by Shahid et al. [36] were not collected during this survey (Table 5). In addition to those species not being present, one new record was collected—*Pr. terraenovae* (74 adults)—with collections occurring across all seasons except the winter. Additionally, *Ch. megacephala* (23 adults) were collected in summer and fall, producing an earlier record than previously recorded by Owings et al. [51].

Owings et al. [51] documented the colonization of human remains by Ch. megacephala at the ARF. In the fall of 2020, larval samples of Ch. megacephala were collected from one donor, and adults were collected from another donor [51]. While relatively few samples (*n* = 7) were collected [51], this study confirmed that Ch. megacephala populations had been present in Tennessee as early as 2018, with most of the specimens collected in the fall (*n* = 22), and only one specimen collected in the summer.

Differences in the species composition between the current study and previously published works may have resulted from differences in the methods used, such as between baited traps and the presence of carrion or cadavers. Passive trapping methods, such as the bait trap method employed in the current study, are now widely used in forensic entomology research [7,8]. A variety of methods have historically been used in research, including sticky traps [19], Schoenly traps [19], and bait bins [52]. Small bait traps are often employed to determine the blowfly community present in a given area. For example, Weidner et al. [9] found that bait traps in New Jersey were an accurate indicator of the main species colonizing remains, since the predominant colonizers (L. sericata, L. coeruleiviridis, and P. regina) of piglet remains were the most numerous flies captured in the bait traps. However, one individual of one species (Pr. terraenovae) was collected from a sweep over the remains, and was not captured in the baited traps [9]. In this study, the differences in the bait (pork kidney vs. beef liver in Weidner et al. [9]) and experimental design (close proximity of the traps to human remains) may explain the capture of Pr. terraenovae in the traps. Additionally, Sanford [19] used passive sticky traps placed at death investigation scenes to collect specimens and compare them to the larvae collected from the decedent’s body. Of the specimens collected, 65% of the cases had one common species in both collection methods, whereas 95% of the cases had at least one species that was unique to a trapping method. Thus, Sanford’s results [18] suggest that collection methods may provide different compositions of forensically relevant specimens associated with decomposition. More recently, LeBlanc et al. [10] examined carcasses’ and bait traps’ species composition, finding that there was a difference in the assemblages and richness of flies in each method. The baited traps disproportionately represented species that could not compete with those found on carcasses, and underrepresented Diptera that were not Calliphoridae.

This study presents with several limitations in relation to fly capture and identification. Our traps were placed in the field for 24 h, regardless of season or temperature. Although our traps were placed in the field for this duration, we employed numerous traps throughout the site to increase potential collections. Furthermore, having the traps surrounded by human remains of varying decomposition stages reduced the chance of only capturing early colonizers. Rather, any blowfly species present on site could be caught. Of the flies captured, 24 could not be morphologically identified down to the species level due to damage to the specimen. Based on the limited morphological information we could obtain from these specimens, we do not believe any species not previously listed was missed. Molecular identifications were not completed during this study due to budgetary constraints, but could be used in future analyses if needed. Finally, this survey was conducted for one year only, allowing for one collection per season, as opposed to a multiyear survey where comparisons could be made across years within season.

Overall, this survey provides baseline information on the forensically relevant blowfly species in the ARF of Knoxville, Tennessee. The ARF is one of the few places where blowfly activities directly associated with human decomposition can be assessed. For this reason, abundant forensic entomological research has taken place there in the past 40 years. In this regard, the findings of this study have potential benefits both practically and academically. In other words, this information can be used to determine which blowflies should be a focus for further research, as well as those that could be expected in criminal investigations. Although flies were taken from baited traps, they were in the vicinity of decomposing human remains, which would have produced larger olfactory cues for the insects to detect, making key species available in the area. This survey found a new species (*Pr. terraenovae*) to be present in the area. Their presence does not necessarily mean that they are dominant colonizers or are established in this area, but should be considered in future research and investigations. This survey also provides baseline information for surrounding states where forensic entomological knowledge is limited.

## Figures and Tables

**Table 1:** Taphonomy facilities found globally *.

Institution	City/Country	Establishment Year
ARF/University of Tennessee	Knoxville, TN, USA	1980
FOREST/Western Carolina University	Cullowhee, NC, USA	2007
FARF/Texas State University	San Marcos, TX, USA	2008
STAFS/Sam Houston State University	Huntsville, TX, USA	2008
CFAR/Southern Illinois University	Carbondale, IL, USA	2012
FIRS/Colorado Mesa University	Grand Junction, CO, USA	2013
AFTER/University of Technology Sydney	Yarramundi, New South Wales, Australia	2016
ARISTA/Amsterdam’s Academic Medical Center	Amsterdam, Netherlands	2017
FIRST/University of South Florida	Tampa, FL, USA	2018
FROST/Northern Michigan University	Marquette, MI, USA	2018
REST[ES]/University of Québec–Trois Rivières	Québec, Canada	2019

* The information for this table was gathered from the published literature [32,33,34].

**Table 2:** Weather data collected during the survey, including the means and ranges of temperatures and humidity when traps were deployed, total precipitation when traps were deployed, and monthly precipitation.

Dates of Trap Deployment and Collection	Temperature (°C)		Humidity (%)		Precipitation (mm)	
	Average	Range	Average	Range	Trap	Monthly
3/4–3/5/18 *	13.9	10.9–14.3	33.3	32.0–40.0	0	144.5
3/19–3/20/18	15.7	12.3–21.6	83.2	51.0–99.0	8.4	
4/4–4/5/18	8.3	1.3–15.3	49.3	25.0–79.0	10.9	108.7
4/19–4/20/18	9.0	2.6–17.1	60.4	32.0–92.0	0	
5/4–5/5/18	22.8	18.1–29.0	70.8	46.0–95.0	0	87.6
5/19–5/20/18	24.7	19.1–30.9	78.1	48.0–99.0	23.1	
6/4–6/5/18	21.2	15.9–28.2	64.0	36.0–89.0	0.5	107.2
6/19–6/20/18	27.5	22.3–34.9	70.1	45.0–90.0	0	
7/4–7/5/18	27.9	23.4–33.1	74.1	58.0–89.0	0	127.5
7/19–7/20/18	25.7	21.2–32.3	77.2	53.0–92.0	0	
8/3–8/4/18	23.8	20.8–29.6	89.7	66.0–96.0	39.6	118.9
8/19–8/20/18	25.6	23.5–30.1	88.1	68.0–95.0	0.5	
9/2–9/3/18	25.6	21.3–37.1	82.0	43.0–97.0	25.4	191.0
9/20–9/21/18	27.1	22.2–39.5	73.1	37.0–93.0	0	
10/4–10/5/18	24.5	20.4–33.3	83.5	53.0–98.0	0	76.2
10/18–10/19/18	12.6	6.7–22.7	75.1	34.0–99.0	0	
11/3–11/4/18	9.3	3.8–18.1	78.8	44.0–99.0	0	142.2
11/17–11/18/18	8.2	1.4–18.1	88.8	43.0–99.0	0	
12/6–12/7/18 ^†^	5.6	3.6–11.2	59.4	36.0–77.0	0	189.5
12/19–12/20/18 ^†^	7.3	4.6–14.3	81.1	46.0–99.0	18.0	
1/3–1/4/19 ^†^	9.5	8.4–12.0	98.3	90.0–99.0	21.6	136.4
1/19–1/20/19 ^†^	5.5	−2.7–12.4	94.9	79.0–99.0	40.1	
2/4/–2/5/19 ^†^	11.7	8.1–18.0	88.6	70.0–99.0	1.5	325.9
2/20–2/21/19 ^†^	9.2	8.6–10.6	98.9	94.0–99.0	47.4	
3/4–3/5/19 ^†^	−1.0	−5.6–7.6	64.1	25.0–86.0	38.6	109.2
3/19–3/20/19 ^†^	9.1	−1.0–25.9	51.2	13.0–93.0	0	
4/4–4/5/19	18.1	10.7–29.9	59.6	19.0–99.0	14.9	111.5
4/20–4/21/19	10.4	7.2–25.4	87.0	31.0–99.0	2.5	

* Weather data were only collected for 3/5/18. ^†^ No insects were collected.

**Table 3:** Number of blowflies collected by season. Percentages for each category are included in parentheses.

Genus	Species *	Overall Totals (%)	Spring (%)	Summer (%)	Fall (%)
**Calliphora**		**110 (3.5%)**	**66 (4.6%)**	**20 (1.5%)**	**24 (6.4%)**
	*C. livida*	15 (0.5%)	13 (0.9%)	1 (0.1%)	1 (0.3%)
	*C. vicina*	63 (2.0%)	38 (2.7%)	18 (1.3%)	7 (1.9%)
	*C. vomitoria*	32(1.0%)	15 (1.1%)	1 (0.1%)	16 (4.3%)
**Chrysomya**		**25 (0.8%)**	**-**	**1 (0.1%)**	**24 (6.4%)**
	*Ch. megacephala*	23 (0.7%)	-	1 (0.1%)	22 (5.9%)
	*Ch. rufifacies*	2 (0.1%)	-	-	2 (0.5%)
**Cochliomyia**		**21 (0.7%)**	**3 (0.2%)**	**18 (1.3%)**	**-**
	*Co. macellaria*	21 (0.7%)	3 (0.2%)	18 (1.3%)	-
**Cynomya**		**2 (0.1%)**	**1 (0.1%)**	**-**	**1 (0.3%)**
	*Cy. cadaverina*	2 (0.1%)	1 (0.1%)	-	1 (0.3%)
**Lucilia**		**844 (26.7%)**	**211 (14.8%)**	**401 (29.5%)**	**232 (61.8%)**
	*L. coeruleiviridis*	649 (20.6%)	138 (9.7%)	290 (21.3%)	221 (58.9%)
	*L. cuprina*	3 (0.1%)	-	-	3 (0.8%)
	*L. illustris*	170 (5.4%)	72 (5.1%)	96 (7.1%)	2 (0.5%)
	*L. sericata*	22 (0.7%)	1 (0.1%)	15 (1.1%)	6 (1.6%)
**Phormia**		**2080 (65.9%)**	**1119 (78.6%)**	**870 (64.0%)**	**91 (24.2%)**
	*P. regina*	2080 (65.9%)	1119 (78.6%)	870 (64.0%)	91 (24.2%)
**Protophormia**		**74 (2.3%)**	**22 (1.5%)**	**49 (3.6%)**	**3 (0.8%)**
	*Pr. terraenovae*	74 (2.3%)	22 (1.5%)	49 (3.6%)	3 (0.8%)

* *Calliphora livida: C. livida; Calliphora vicina: C. vicina; Calliphora vomitoria: C. vomitoria; Chrysomya megacephala: Ch. megacephala; Chrysomya rufifacies: Ch. rufifacies; Cochliomyia macellaria: Co. macellaria; Cynomya cadaverina: Cy. Cadaverine; Lucilia coeruleiviridis: L. coeruleiviridis; Lucilia cuprina: L. cuprina; Lucilia illustris: L. illustris; Lucilia sericata: L. sericata; Phormia regina: P. regina; Protophormia terraenovae: Pr. terraenovae*.

**Table 4:** Species richness and Simpson’s index of diversity by season.

Season	Richness	Simpson’s Index of Diversity
Spring	10	0.368
Summer	10	0.538
Fall	12	0.589
Winter	0	-

**Table 5:** Comparison of species collected previously at the ARF to the present study. A + symbol and shading indicates the presence of that species, while a – symbol indicates its absence.

Species *	Reed [50]	Shahid et al. [36]	Schoenly et al. [41]	Present Study
*C. livida*	+	–	–	+
*C. vicina*	+	+	–	+
*C. vomitoria*	+	–	–	+
*C. terraenovae*	+	–	–	–
*Ch. megacephala*	–	–	–	+
*Ch. rufifacies*	–	+	–	+
*Co. macellaria*	+	+	+	+
*Cy. cadaverina*	+	–	–	+
*L. coeruleiviridis*	+	+	+	+
*L. cuprina*	+	–	–	+
*L. illustris*	+	+	+	+
*L. sericata*	+	+	+	+
*L. cluvia*	–	+	–	–
*P. regina*	+	+	+	+
*Pr. terraenovae*	–	–	–	+

* *Calliphora livida: C. livida; Calliphora vicina: C. vicina; Calliphora vomitoria: C. vomitoria; Calliphora terraenovae: C. terraenovae; Chrysomya megacephala: Ch. megacephala; Chrysomya rufifacies: Ch. Rufifacies; Cochliomyia macellaria: Co. macellaria; Cynomya cadaverine: Cy. Cadaverine; Lucilia coeruleiviridis: L. coeruleiviridis; Lucilia cuprina: L. cuprina; Lucilia illustris: L. illustris; Lucilia sericata: L. sericata; Lucilia cluvia: L. cluvia; Phormia regina: P. regina; Protophormia terraenovae: Pr. terraenovae*.

## Data Availability

Data supporting the conclusions of this article are included within the article. The datasets used and/or analyzed during the present study are available from the corresponding author upon reasonable request.
